# What makes working together work? A scoping review of the guidance on North–South research partnerships

**DOI:** 10.1093/heapol/czac008

**Published:** 2022-01-28

**Authors:** Shirine Voller, Joanna Schellenberg, Primus Chi, Nicki Thorogood

**Affiliations:** Faculty of Public Health and Policy, London School of Hygiene & Tropical Medicine, 15-17 Tavistock Place, London WC1H 9SH, UK; Epidemiology and Demography Department, KEMRI Wellcome Trust Research Programme, CGMRC, PO Box 230-80108 Kilifi, Kenya; Faculty of Infectious and Tropical Diseases, London School of Hygiene & Tropical Medicine, Keppel Street, London WC1E 7HT, UK; Health Systems and Research Ethics Department, KEMRI Wellcome Trust Research Programme, CGMRC, PO Box 230-80108 Kilifi, Kenya; Faculty of Public Health and Policy, London School of Hygiene & Tropical Medicine, 15-17 Tavistock Place, London WC1H 9SH, UK

**Keywords:** North–South research partnerships, principles, guidelines, scoping review

## Abstract

At their best, research partnerships provide a mechanism to optimize each partner’s strengths, make scientific discoveries and achieve development goals. Each partner stands to gain from the relationship and perceives it to be fair. However, partnerships between institutions in the global North and the global South have been beleaguered by structural inequalities and power imbalances, and Northern stakeholders have been criticized for perpetuating paternalistic or neo-colonial behaviours. As part of efforts to redress imbalances and achieve equity and mutual benefit, various principles, guidelines, frameworks and models for partnership have been developed. This scoping review maps the literature and summarizes key features of the guidelines for North–South research partnerships. The review was conducted between October 2020 and January 2021. Three academic journal databases and Google were searched, and additional resources were identified through a hand search of reference lists and expert recommendation. Twenty-two guidelines were identified published between 1994 and 2021 and originating predominantly in the fields of international development and global health. The themes addressed within the guidelines were aggregated using NVivo qualitative analysis software to code the content of each guideline. Topics featuring most prominently in the guidelines were: partner roles, responsibilities and ways of working; capacity strengthening; motivation and goals; resource contributions; agenda setting and study design; governance structures and institutional agreements; dissemination; respect for affected populations; data handling and ownership; funding and long-term commitments. The current study reinforces many of the themes from two recent scoping reviews specific to the field of global health, but gaps remain, which need to be addressed: Southern stakeholders continue to be under-represented in guideline development, and there is limited evidence of how guidelines are used in practice. Further exploration is needed of Southern stakeholder priorities and whether and how guidelines are operationalized.

**Key messages**

- There are many sources of guidance for North–South research partnerships, predominantly from the fields of global health and international development.
- There is high concordance within existing guidance on the themes and principles for achieving equity in North–South research partnerships.
- Stakeholders from the global South are under-represented in guideline development, and more work is needed to understand all partners’ priorities for partnership.
- There is limited evidence of whether and how guidelines are used in practice.

## Introduction

Partnership is seen as an important mechanism for improving health and achieving development goals (United Nations, 2020). It is often associated with a set of values such as responsibility, joint decision-making, trust and mutual understanding (Mommers and van Wessel 2009; Corbin *et al.*, 2012) and has been characterized as a ‘cooperation strategy…governed by a comprehensive and inclusive perspective…and promoting synergetic actions and initiatives’ (Forti, 2005, p. 32). Costello and Zumla (2000) advocated for an emerging model of partnership research in low- and middle-income countries (LMICs) over its predecessor, which they termed the ‘semi-colonial’ model. They described partnerships as having, amongst other characteristics, a jointly negotiated research agenda, integral links with national institutions, nationally led line management, strong influence on local policy makers, dissemination balanced between international, national and regional journals and a role in strengthening national academic infrastructure. They contrasted this with the semi-colonial model in which the research agenda is dominated by outsiders, only peripheral links are established with national institutions, dissemination is focused on international outputs and there is little engagement of local policy makers (p. 828). Notably, even while promoting the emerging model of partnership, the language that Costello and Zumla used was entrenched in assumptions: ‘local’ and ‘national’ were used to refer to LMIC institutions, while ‘international’ largely referred to high-income country institutions. These, among other terms, remain prevalent in the discourse of global health (itself a questionable term), which matters because language both influences and reveals attitudes towards colonial roots (Hommes *et al.*, 2021). The terms North and South are also imperfect and crude but remain sufficiently widespread that they were felt to be appropriate to use in this scoping review.

Examples of ‘mosquito researchers’ and ‘parachutists’ (Edejer, 1999, p. 2) from the global North, who take data and samples for analysis and writing up and make little effort to share results with the community in which the research was conducted, (Edejer, 1999; Binka, 2005; Craveiro *et al.*, 2020) are, thankfully, rarely reported in recent literature. However, partnerships are not a panacea and can disguise ‘insidious subversive ill effects’ (Edejer, 1999, p. 439). These include one-way accountability, transparency and reporting, whereby Northern partners, often the prime grant recipient, place extensive demands on sub-contracted partners while having less scrutiny of their own processes of operation (Harrison, 2002; Matenga *et al.*, 2019).

Northern partners are often disproportionately advantaged over their Southern collaborators in terms of access to resources, including funding, knowledge, expert networks and education and development opportunities, and typically have greater power and influence in all facets of the relationship (Healey-Walsh *et al.*, 2019; Craveiro *et al.*, 2020). Unequal power relations have also be seen in the way in which research agendas are set, whereby Northern funders and donors frame research topics (Binka, 2005; Viergever *et al.*, 2010; Franzen *et al.*, 2017; Bradley, 2017), which may not reflect priorities at Southern partner sites (Coloma and Harris 2009; Kunert *et al.*, 2020). An imperative to secure funds compounded by unclear institutional research priorities have been cited as factors contributing to the weak position in agenda setting that Southern partners have historically occupied (Bradley, 2008). Northern research institutions and funders have also been dominant in determining which partners to approach and what benefits they receive (Bradley, 2008; 2007; White, 2007). In some instances, Southern partners are involved in a tokenistic way at the application stage if a funding call requires a Southern collaborator to be named (Murphy *et al.*, 2015; Gautier *et al.*, 2018), and may only be invited on board, and even then may not feel fully involved, once the direction and scope of work have been decided (Forti, 2005). Unequal power dynamics extend to Northern partners frequently leading programmes of research, setting ethical standards and managerial rules (Gautier *et al.*, 2018), governing the partnership’s administration and budget management (Gaillard, 1994; Carbonnier and Kontinen 2014; Murphy *et al.*, 2015; Matenga *et al.*, 2019) and even instigating the creation of parallel structures that bypass local institutions (Sawyerr, 2004). Southern partners may be confined to operational roles (Mony *et al.*, 2005) such as fieldwork coordinators (Craveiro *et al.*, 2020) in what has been described as a relational structure of ‘subordinate integration’ (Feld and Kreimer 2019, p. 166). The dominance of Northern researchers in academic authorship has been attributed to their senior positions in the partnership hierarchy and Southern researchers’ lack of experience in scientific writing compounded by the conventions of academic publishing, whereby certain types of contribution are privileged over others (Mony *et al.*, 2005; Walsh *et al.*, 2016; Gautier *et al.*, 2018; Craveiro *et al.*, 2020; González-Alcaide *et al.*, 2020).

Further challenges exist due to the way in which global health research is incentivized, and its success judged. Research funds typically operate on short-term project lifecycles which attribute value to research outputs over the fulfilment of principles of partnership, and criteria for academic promotion do not explicitly reward long-term commitment between Northern and Southern partners (Bradley, 2008), nor the policy-oriented, applied research outputs often arising from partnerships (RAWOO, 2001). More diverse indicators of success are increasingly being applied, including sustainability of interventions and investment in research capacity development (Edejer, 1999), and there is some evidence that traditional hierarchies of authorship are shifting to award greater recognition to the contribution of those leading field research activities (White, 2020).

In sum, a range of structural inequalities and historical legacies in the relationship between the global North and South (Bradley, 2007; Craveiro *et al.*, 2020) remain central to the challenges of realizing partnership ideals of equity and mutual benefit (Jentsch and Pilley 2003). Critics have argued that the benefits of partnership have been channelled disproportionately to the global North (Bradley, 2007; Crane, 2010), and there is a need to redress the balance. Furthermore, the philosophical underpinnings of global health are increasingly being scrutinized. There are calls for a fundamental re-formulation of the systems, structures and attitudes that sustain global health, and growing pressure to decolonize the field. As these discussions gain momentum and stimulate change within the system of global health, efforts also are being made at an operational level to work towards equity through the development and application of principles and guidelines for partnerships.

For practitioners working in global health who want to assess and improve their partnership practice, navigating the guidance on partnerships can be overwhelming. This scoping review aims to offer assistance by identifying, characterizing and summarizing a broad range of published guidance on North–South research partnerships, searching beyond the field of global health to accommodate guidance from other fields. It includes principles for how partners should behave, guidelines for operationalizing research partnerships and frameworks and models that characterize the components of equitable North–South research partnerships. Empirical studies yield valuable lessons for practice and are essential to illustrate the challenges that project teams face and strategies employed in pursuit of fulfilling partnership goals but were outside of the scope of this review.

This review seeks to complement the findings of two scoping reviews specific to global health, which were published in early 2021 (Faure *et al.*, 2021; Monette *et al.*, 2021) when our review was completed, and explores whether extending the search beyond global health to other fields of research yields fresh perspectives on effective partnership working.

The review is intended to help practitioners navigate the extensive guidance available and identify what to focus on to improve how the North–South partnerships they are involved in work. Practitioners from the global South are particularly encouraged to critique the review’s findings and consider whether there are gaps in the existing guidance that need to be addressed.

## Methods

Scoping reviews typically seek to achieve some or all of the following objectives: to identify the types of evidence available in a given field; to clarify key concepts or definitions in the literature; to examine how research is conducted on a certain topic or field; to identify key characteristics relating to a concept as a precursor to a systematic review and to identify and analyse knowledge gaps (Munn *et al.*, 2018). Our scoping review substantially addresses the following objectives: ‘to identify the types of available evidence in a given field’ and ‘to identify key characteristics or factors related to a concept’. In both objectives, we aim to look beyond the global health literature to also explore evidence in other fields (e.g. international development), hence consolidating evidence on equity in partnerships that can be utilized beyond the field of global health. Scoping reviews are also used to report on evidence that informs practice and in emerging fields of study (Munn *et al.*, 2018). Since the field of research partnership guidelines spans the academic and practitioner domains and attention on North–South partnerships has grown in recent decades, this reinforced the utility of the scoping review methodology. The steps of Arksey and O’Malley’s methodological framework for conducting scoping reviews (Arksey and O’Malley 2005) were broadly followed:

### Step 1: identifying the research question

The question addressed by the review was *‘What are the characteristics of the principles, guidelines, frameworks and models which have been developed to guide the*  *operationalization*  *of North*–*South research partnerships?’*

### Steps 2 and 3: identifying relevant studies and study selection

Relevant studies were identified by searching three academic journals databases, PubMed, Scopus and Web of Science, between 26 October and 16 November 2020 applying the search string:

(North–South) AND research AND (Partnership OR Collaboration) AND (guideline OR principle OR framework OR model).

No date filters were applied to the search. Titles and abstracts were screened for relevance, and a check was made on whether the article was freely available in full text and whether it was written in English. Once articles had been discarded that did not satisfy these requirements and duplicates were removed, the remaining articles were read in full to identify principles, guidelines, frameworks and models for partnerships (hereafter referred to collectively as ‘guidelines’) either directly or from references cited within the article. Guidelines were included where they were derived from a broad body of experience, evidence or both. Guidelines were excluded where they emerged as lessons learned from an empirical study of an individual project partnership. Write-ups of individual project partnerships are valuable for illustrating challenges faced and strategies successfully used by stakeholders to achieve the goals of their partnership and can generate valuable guidance, which may be generalizable to other situations but fell outside the scope of this review. A complementary search to the database search was conducted in Google in December 2020. The top 100 hits, not including sponsored links, were reviewed for relevance. Duplicates were removed, and the full text of the remaining publications was reviewed to identify guidelines for inclusion.

In Arksey and O’Malley’s methodological framework (Arksey and O’Malley 2005), *Consultation* is included as an optional sixth step, while in this review, it was incorporated as part of the study identification and selection process.

### Step 4: charting the data

A data charting form was developed to extract data about descriptive characteristics of each guideline and guideline content. The form included fields for: output type, field of research or implementation, target audience and methodology by which guideline was developed and key features of the guideline.

### Step 5: collating, summarizing and reporting results

Two steps were followed in collating, summarizing and reporting the results. First, descriptive characteristics were extracted and listed in the data charting form. An iterative process of deriving categories for each characteristic was undertaken, whereby data from the data charting forms were aggregated in an excel spreadsheet and categories developed from the data for each characteristic of interest. Second, guidelines were uploaded in full text into NVivo to facilitate inductive coding of the topics addressed by the guidelines. Once all guidelines had been coded, the codes were reviewed, revised and organized within NVivo.

## Results

### Identifying guidelines

The database searches returned a total of 1224 articles. One thousand one hundred and forty-seven articles were discarded at the title and abstract review stage because they were not relevant, were not available in full text or were not available in English. Of the remaining 77 articles, 24 were duplicates and were removed, leaving 53 unique articles across the three databases. Two guidelines were identified directly from these articles, and a further nine from reference lists.

The Google search yielded 47 relevant publications. Once duplicates were removed, 32 unique publications remained, from which five new guidelines were identified. A further six guidelines were identified from wider reading and recommendations during December 2020 and January 2021.

**Figure 1.**
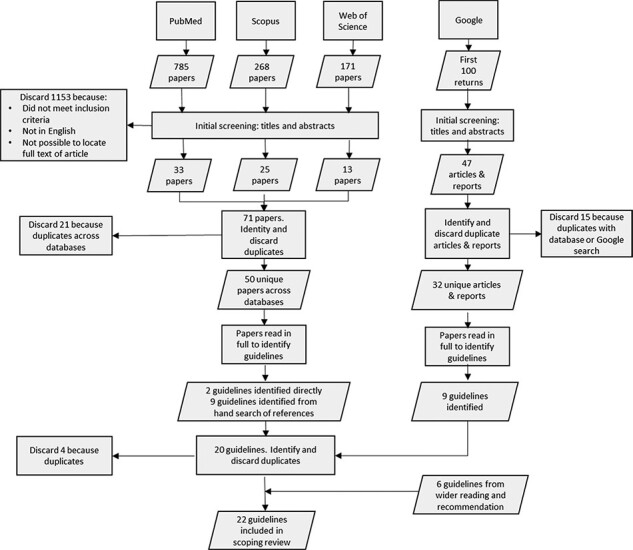
Modified PRISMA (Page *et al.*, 2021) flow diagram depicting scoping review study identification and selection

In total, 224 documents were selected for consideration in the review. Figure 1 depicts the identification and selection process, while Table 1 lists the guidelines selected for inclusion.

**Table 1.** Partnership guidelines included in the scoping review

	Author and date of publication	Guideline name	Publication type	Field
1	Afsana *et al.*, 2009	Partnership Assessment Toolkit	Toolkit	Global Health
2	Alba *et al.*, 2020b	Bridging research integrity and global health epidemiology (BRIDGE) guidelines	Journal article	Global Health
3	Association of Universities and Colleges of Canada, 2013	Three sets of characteristics of effective and innovative partnerships	Report	International development
4	Canadian Coalition for Global Health Research, 2015	CCGHR principles for global health research	Guideline	Global Health
5	Carbonnier and Kontinen 2014	North–South Research Partnership, Academia meets Development	Policy brief/ report	International development
6	Cornish *et al.*, 2017	Rethinking research partnerships	Toolkit	International development
7	Costello and Zumla 2000	Moving to research partnerships in developing countries	Journal article	Global health
8	Dodson, 2017	Ten ways in which funders can influence equitable partnerships	Report	International development
9	Ecosystem Services for Poverty Alleviation ESPA Directorate, 2018	Three constituent factors of equitable partnerships	Policy brief	International development
10	Faure *et al.*, 2021	Ten key areas for developing equitable international collaborations	Journal article	Global Health
11	Gaillard, 1994	Charter of North South partners	Journal article	International development
12	Kennedy *et al.*, 2006	Ten steps in the process of ethical research collaboration across ethnically and culturally diverse communities	Journal article	Midwifery/ Integrity and ethics
13	Larkan *et al.*, 2016	Attributes and derived core concepts for successful research partnerships in global health	Journal article	Global Health
14	Leffers and Mitchell 2011	Conceptual Model for Partnership and Sustainability in Global Health	Journal article	Global Health
15	3rd World Conference on Research Integrity, 2013	Responsibilities of Individual and Institutional Partners in Cross-Boundary Research Collaborations	Guideline	Integrity and ethics
16	Newman *et al.*, 2019/ Rethinking Research Collaborative, 2018	Eight principles for fair and equitable research partnerships	Journal article and linked report	International development
17	Migot-Adholla and Warner, 2005	Five characteristics of successful North South Partnerships	Guideline	International development
18	RAWOO, 1999	Three principles for a fruitful partnership	Report	International development
19	Research Fairness Initiative, 2018	Three domains, five topics per domain and three indicators per topic	Toolkit	Global health
20	Stöckli *et al.*, 2018	11 principles and 7 questions	Toolkit	International development
21	Taylor and Berg 2019	Seven steps for developing trust	Journal article	Global health
22	Trust, 2018	Global Code of Conduct for Research in Resource-Poor Settings	Guideline	Research

### Publication date

The earliest guideline was published in 1994 and the most recent in January 2021. The majority of guidelines (*n* = 15) were published or last updated in the decade 2011–2020; more than twice as many were published in the previous decade (*n* = 7). Only two guidelines were published before 2000.

### Field of origin

Most guidelines for research partnerships originated from and were targeted towards two broad fields: international development (*n* = 10) and global health (*n* = 10). Two guidelines emerged from the field of research integrity and ethics (3rd World Conference on Research Integrity, 2013; Trust, 2018).

### Output type

Guidelines were published in a variety of forms. The predominant output type was academic journal articles (*n* = 9). Other output types were reports (*n* = 4), guidelines (*n* = 4), toolkits (*n* = 4), websites (*n* = 3) and policy papers (*n* = 2). In several cases, guidelines were substantiated by a package of supporting information or in multiple formats. For example, the Swiss Commission for Research Partnerships with Developing Countries (KFPE) 11 principles and seven questions guide (Stöckli *et al.*, 2018) existed as a downloadable pdf supported by web-based resources, and the Council on Health Research for Development’s Research Fairness Initiative (RFI) (2018) offered three versions of the RFI guide on its website alongside examples of institutional self-assessments and links to supporting resources and additional information. Newman *et al.* (2019) described eight principles for fair and equitable research partnerships in the Institute for Development Studies bulletin, which were also detailed in a report by the Rethinking Research Collaborative (2018). The Bridging Research Integrity and Global Epidemiology (BRIDGE) guidelines featured in two journal articles (Alba *et al.*, 2020a,b), and a website provided supporting material.

### Target audience

About a quarter (*n* = 5) of guidelines did not explicitly describe their target audience. Where one or more audiences were specified, the most common categories were researchers (*n* = 12), funders (*n* = 11) and a catch-all category of ‘all other stakeholders’ (*n* = 8). INGOs, health professionals, government agencies, policy makers, civil society, research administrators and students were each mentioned in between one and four guidelines. Only two guidelines were targeted towards a narrow audience: Dodson (2017) focused on the role of funders in equitable and effective international development collaborations, while Kennedy *et al.* (2006) designed their guideline for midwives, although the authors commented that it could be used by researchers in other fields. No guidelines explicitly articulated a distinction between audiences in the global North and the global South.

### How guidelines were developed

A range of research methodologies and consultative techniques were used to inform guideline development. Half (*n* = 11) of guidelines were informed by existing literature. Faure *et al.* (2021) used a scoping review to identify 10 key areas for equitable partnership, drawing largely on qualitative empirical studies, while other authors used a literature review in combination with empirical research. For example, Alba *et al.* (2020b) drew on the literature from two domains: best practices in epidemiology and research fairness to develop provisional guidelines, which they tested and refined using a Delphi consultation to create the BRIDGE guidelines. The Association of Universities and Colleges of Canada (AUCC) developed an analytical framework (Association of Universities and Colleges of Canada, 2013) based on literature, which informed the design of data collection and analysis tools that they used to assess a number of partnership case studies. Two guidelines used the KFPE principles (Stöckli *et al.*, 2018) as a starting point: Costello and Zumla (2000) used them to inform a checklist of questions to consider, and Migot-Adholla and Warner (Migot-Adholla and Warner 2005) integrated them with personal experience to identify five characteristics of successful partnerships. Most guidelines (*n* = 15) were developed using multiple methods, while seven guidelines were developed based on a single method.

A third of guidelines (*n* = 8) used round table discussions and workshops to generate data, and over a quarter (*n* = 7) used interviews. All five studies that employed surveys did so in combination with at least one other data collection method. Four guidelines documented their use of stakeholder consultation (Research Fairness Initiative 2018; Stöckli *et al.*, 2018; Trust, 2018; Taylor and Berg 2019). In four guidelines, the authors described drawing on their personal experience as practitioners (Costello and Zumla 2000; Migot-Adholla and Warner 2005; Leffers and Mitchell 2011; Taylor and Berg 2019) and in two guidelines documents pertaining to particular North–South partnerships were analysed (Gaillard, 1994; ESPA Directorate, 2018). Two guidelines were developed using a grounded theory approach: Leffers and Mitchell (2011) interviewed 13 global health nurse experts and compared empirical findings with themes from the literature to develop a model of partnership and sustainability in global health, while Larkan *et al.* (2016) used a questionnaire and consultative meetings to develop a unifying framework for partnership.

Stakeholders from the global North featured more prominently as participants in the research and consultations, which led to guideline development than stakeholders from the global South (see Table 2). Ten guidelines were developed predominantly or exclusively drawing on Northern stakeholders as participants, while only two were developed predominantly or exclusively drawing on Southern stakeholders as participants. In almost a third of guidelines (*n* = 7), it was not clear from the methods described within the guideline what the balance was of Northern and Southern participants who contributed to guideline development.

**Table 2.** Geographic location of participants contributing to guideline development

Stakeholder geographic location	Number of guidelines
More North than South	7
Not specified	6
Equal balance of North and South	3
All North	3
All South	1
Mix of North and South—balance unspecified	1
More South than North	1

### Guideline structure

A number of guidelines were structured as representations of the research partnership lifecycle. For example, the Canadian Coalition for Global Health Research’s Partnership Assessment Toolkit (Afsana *et al.*, 2009) comprised four phases: *Inception, Implementation, Dissemination* and ‘*Good endings and new beginnings*’. The AUCC organized their guideline (Association of Universities and Colleges of Canada, 2013) into items under three headings: *foundational principles, sustaining processes* and *results and activities*, and the Research Fairness Initiative (2018) identified five topics and three indicators per topic within the domains of *fairness of opportunity* before research starts, *fair process* during research and *fair sharing of benefits, costs and outcomes* at the end of a research partnership. In the Rethinking Research Partnerships report (Cornish *et al.*, 2017), the authors highlighted six phases and structured the report into chapters around these: *understanding the context, establishing the partnership, sustaining the partnership, designing and implementing research, communicating and ensuring impact* and *beyond the partnership*, while Alba *et al.’s* (2020a) comprised six standards, or phases, of the research process. The phases were: *study preparation*, *protocol development, data collection, data management, data analysis* and *dissemination and communication.* The KFPE principles (Stöckli *et al.*, 2018) partially mapped onto the research partnership lifecycle. The principles were: *set the agenda together, interact with stakeholders, clarify responsibilities, account to beneficiaries, promote mutual learning, enhance capacities, share data and networks, disseminate results, pool profits and merits, apply results* and *secure outcomes.* Each principle was accompanied by a description of the issues within the principle, the main challenges in upholding it and a checklist of steps to follow when applying the principle.

Taylor and Berg’s guideline for partnership (Taylor and Berg 2019) focused on trust, and they articulated seven steps to developing trust. Whilst not mapped directly to the research lifecycle, these steps offered a set of consecutive instructions to follow. Kennedy *et al.* (2006) also used the concept of steps, describing 10 steps in the process of ethical research collaboration across ethnically and culturally diverse communities.

Guidelines were further categorized as *values*  *based*, *activity*  *based* or *combined values*  *and activity*  *based*. An example of a values-based guideline was the Global Code of Conduct for Research in Resource-Poor Settings (Trust, 2018), which was organized into four domains: *fairness, respect, care* and *honesty* with four to eight articles within each domain. The Canadian Coalition for Global Health Research (2015) also followed a values-based approach. The authors identified six principles, all linked to a core concept of equity. These were: *authentic partnering, inclusion, shared benefits, commitment to the future, responsiveness to the causes of inequity* and *humility*. Newman *et al.*’s eight principles for fair and equitable research partnerships (Newman *et al.*, 2019) were predominantly values based and addressed the following issues: *put poverty first, critically engage with context(s), redress evidence hierarchies, adapt and respond, respect diversity of knowledge and skills, commit to transparency, invest in relationships* and *keep learning.*

Activity-based guidelines were organized around concrete actions or topics. For example, the Netherlands Development Assistance Research Council (RAWOO, 1999) proposed three conditions for a fruitful partnership: a *broad based consultative process should precede any programme, the Northern partner should be prepared to relinquish control and accept considerable Southern partner autonomy* and *capacity strengthening should be a specific aim of the partnership*. The KFPE principles (Stöckli *et al.*, 2018), Dodson’s guidelines for funders (Dodson, 2017) and the Overseas Development Institute guidelines (Migot-Adholla and Warner 2005) were also categorized as activity based. The Montreal Statement on Research Integrity (3rd World Conference on Research Integrity, 2013) organized its largely activity-based guideline into four areas of responsibility: *General collaborative responsibilities, responsibilities in managing the collaboration, responsibilities in collaborative relationships* and *responsibilities for outcomes of research.* The statement listed 20 responsibilities divided across these four areas.

Guidelines that combined values and activities included Costello and Zumla (2000) who highlighted the importance of *mutual trust and shared decision-making, development of national research capacity, national ownership* and *emphasis on getting research findings into policy and practice,* and Larkan *et al.* (2016) who derived seven core concepts from a set of attributes for successful global health partnerships. These were: *focus, values, equity, benefit, leadership, communication* and *resolution*. Faure *et al.*’s 10 key areas for developing equitable international collaborations (Faure *et al.*, 2021) and Gaillard’s charter of North–South partners (Gaillard, 1994) also combined values and activities.

One guideline was a model for understanding partnership (Leffers and Mitchell 2011). This conceptual model for partnership and sustainability in global health integrated partner factors or characteristics, key components and processes for partnership development and factors affecting sustainability.

### Key areas of attention for North–South partnerships

The topic areas that partnerships should focus on are summarized in Table 3 below, ranked in order of the number of guidelines, which included a particular area. A total of 21 topic areas were included in two or more guidelines. Supplementary Table S1 shows which topics were included within which guidelines. The top 11 topic areas are discussed below.

**Table 3.** Topics addressed by partnership guidelines

Ranking	Topic	# Guidelines
1	Roles, responsibilities and ways of working	18
2	Capacity strengthening	15
3	Motivation and goals	14
=3	Resource contributions	14
5	Agenda setting and study design	11
=5	Governance structures, institutional agreements	11
7	Dissemination	10
=7	Respect for affected populations, including local relevance	10
=7	Data handling and ownership	10
10	Funding	8
=10	Long-term commitments	8
12	Acknowledging power dynamics and inequalities	7
=12	Trust	7
14	Monitoring and evaluation	6
=14	Ethical approvals	6
=14	Shared benefits	6
17	Justification for research	5
18	Appreciation of context	4
19	Administrative support	2
=19	Closure plans	2
=19	Leadership	2

The topic *roles, responsibilities and ways of working* was present in 18 of the 22 guidelines. This topic encompassed several sub-topics, including *processes to support regular, open and transparent communication between partners* (*n* = 8) and a *commitment to transparency* (*n* = 5), particularly around finance and administration. Several guidelines described the importance of *jointly agreed mechanisms for conflict resolution* (n = 5), while *decision-making* (*n* = 5), *accountability* (*n* = 3) and the *role of brokers* to represent constituent organizations within a partnership and the partnership as a whole (*n* = 3) were also highlighted.

*Capacity strengthening* was the second most prominent topic, featuring in 15 of 22 guidelines. Guidelines differentially emphasized whose capacity was to be strengthened and how, with attention drawn to individual, institutional and systemic or national capacity and focusing both on capacity for research and for research management, including budgeting, contracting and ethics. Some guidelines framed the provision of resources and expertise to support the development of the weaker partner as a fundamental responsibility of the stronger partner in order for partners to collaborate on a more equal playing field. Other guidelines presented capacity strengthening as bi-directional and talked about mutual learning and growth.

The topic *motivation and goals* was addressed in 14 guidelines. Guidelines emphasized the importance of discussing and legitimizing each partner’s respective interests and priorities, as well as identifying mutual benefits and shared goals for the partnership. Balancing individual and joint objectives was seen to be important for the sustainability of a partnership and for developing trust.

The topic r*esource contributions* was also addressed in 14 guidelines. Guidelines emphasized the need to discuss and agree what resources each stakeholder would commit to the partnership and for different types of contribution to be recognized and valued. Several guidelines highlighted the relationship between costs and benefits and suggested that the benefits accrued by each partner should be in proportion to the costs of participating in the partnership.

*Agenda setting and study design* were combined into a single topic that was represented in half (*n* = 11) of the guidelines. This topic emphasized the importance of the research agenda being set jointly, all partners being involved in proposal writing and agreement being reached on study design, especially in multi-sectoral research studies.

The topic *governance structures and institutional agreements* was closely aligned with *roles, responsibilities and ways of working* but was classified as a separate topic because of the number of guidelines (*n* = 11) that specifically mentioned the need for partnership arrangements to be documented in a formal agreement and enacted through governance structures. The types of agreement described included memoranda of understanding, codes of conduct, terms of reference and research agreements.

The topic *dissemination* was identified in 10 guidelines. Issues addressed included the obligation on partnerships to make research findings available in a format appropriate to the audience and for a wide range of audiences to be considered in dissemination plans, including the populations involved in and affected by the research. This topic also included the issue of authorship and the need for expectations and opportunities for authorship to be clear and agreed on by all partners.

The topic *respect for affected populations, including local relevance* was identified in 10 guidelines. The topic overlapped with *dissemination* but went further to include the expectation that research results should be not only be made available in the public domain, but partners should push for the translation of findings into policy and practice. This topic also addressed the imperative for research only to be done where there was buy-in from and relevance to the communities in which it was planned to take place and where it addressed a priority health issue in the country or region.

A number of issues involving research data collection, management, storage, sharing, use and ownership were reflected within the topic *Data handling and ownership* and featured in 10 guidelines. Many of the issues pertained to the need for clear and jointly agreed plans between partners for all data-related issues, with particular emphasis on data ownership and use.

*Funding* featured as a topic in eight guidelines and covered a range of issues, including the need to secure long-term, core funding to achieve sustainability whilst recognizing the typically short, e.g. 3–5 years, time horizon of individual research grants and recognizing the differential funding opportunities available to partners in the global North and global South. Knock-on issues included how funds were channelled to each partner, the need for funds to be fairly distributed between partners, the need for funds to adequately cover the costs of engaging in research and the need to consider the implications of funder-specific rules and requirements on project feasibility. The topic *long-term commitments* was addressed by eight guidelines. As with the topic of funding, several guidelines linked long-term commitments to sustainability and to the elevation of relationships above individual projects towards institutional relationships. The time required to establish and build meaningful relationships at an individual and institutional level was also captured within this topic.

Ten other topics were identified in two or more guidelines. These were: acknowledging power dynamics and inequalities (*n* = 7), trust (*n* = 7), monitoring and evaluation (*n* = 6), ethical approvals (*n* = 6), shared benefits (*n* = 6), justification for research (*n* = 5), appreciation of context (*n* = 4), administrative support (*n* = 2), closure plans (*n* = 2) and leadership (*n* = 2).

### Comparison of topics across disciplines

The topics identified were compared by discipline to establish whether guidelines from the discipline of global health had a substantially different emphasis compared with international development. Table 4 shows the topics disaggregated by discipline. Topics are emboldened where they were more than twice as prevalent in guidelines from one of the disciplines compared to the other. The two guidelines from the field of research integrity and ethics were included in the comparison table, but no attempt was made to compare them against the other disciplines due to the small sample size.

**Table 4.** Topics by discipline

Topics addressed by partnership guidelines	Number of guidelines addressing each topic			
	Total	Global health	Int dev	Integrity & ethics
Roles, responsibilities & ways of working	18	8	8	2
Capacity strengthening	15	7	7	1
Motivation & goals	14	6	6	2
Resource contributions	**14**	**4**	**9**	1
Agenda setting & study design	11	4	7	0
Governance structures, institutional agreements	**11**	**7**	**3**	1
Dissemination	10	5	3	2
Respect for affected populations, including local relevance	**10**	**7**	**2**	1
Data handling and ownership	**10**	**6**	**2**	2
Funding	8	4	3	1
Long term commitments	8	3	5	0
Acknowledging power dynamics and inequalities	**7**	**5**	**2**	0
Trust	**7**	**5**	**1**	1
Monitoring & evaluation	6	2	3	1
Ethical approvals	**6**	**4**	**1**	1
Shared benefits	**6**	**1**	**3**	2
Justification for research	**5**	**3**	**1**	1
Appreciation of context	4	2	2	0
Administrative support	**2**	**2**	**0**	0
Closure plans	2	1	1	0
Leadership	2	1	1	0

The top three topics identified overall: roles, responsibilities and ways of working, capacity strengthening and motivation and goals, featured equally prominently in guidelines from the fields of global health and international development. Two topics featured more prominently in guidelines from international development compared to global health: resource contributions and shared benefits. In contrast, eight topics featured more prominently in guidelines from global health compared to international development: governance structures and institutional agreements; respect for affected populations; data collection, management, storage, sharing, use and ownership; acknowledging power dynamics and inequalities; trust; ethical approvals; justification for research and administrative support.

### Comparing the findings with reviews specific to global health partnerships

Topics identified from this scoping review were compared with the topics identified in scoping reviews from the field of global health conducted by Faure *et al.* (2021) in October–November 2019 and Monette *et al.* (2021) in February 2020. These scoping reviews included 11 and 9 resources, respectively, while the current study included 22 guidelines. There was strong overlap in the themes identified across all three reviews. The 10 topics identified in Faure *et al.*’s review were also reflected within the top 12 topics of this review, while of the 18 principles that featured in at least two of Monette *et al.*’s sources, 14 were also identified in this review (see Table 5).

**Table 5.** Comparison of themes between three scoping reviews on North South research partnerships

Rank	This review	Faure *et al.* Jan (2021)^a^	Monette *et al.* Mar (2021)^b^
1	Roles, responsibilities and ways of working	Communication (10)	Define Roles (2); communication (2); transparency (2)
2	Capacity strengthening	Capacity building (2)	Capacity Building/strengthening (3); Mutual learning (2)
3	Resource contributions		
4	Motivation and goals		
5	Agenda setting and study design		Agenda Setting (3)
6	Governance structures, institutional agreements	Research agreement (5)	Accountability (3)
=6	Dissemination	Authorship (3)	
8	Respect for affected populations, including local relevance	Local health priorities (6); Recognition of stakeholders (9)	Engage stakeholders (2); Actionable research (2)
=8	Data collection, management, storage, sharing, use and ownership	Sample ownership (4)	Data access (2)
10	Funding	Funding (1)	
=10	Long-term commitments		Sustainability (3)
12	Acknowledging power dynamics and inequalities	Acknowledging inequalities (8)	
=12	Trust	Trust (7)	Trust (2)
14	Monitoring and evaluation		
15	Ethical approvals		
=15	Shared benefits		Mutual Benefits (6)
17	Justification for research		
18	Appreciation of context		Understand the context (2)
19	Administrative support		
20	Closure plans		
=20	Leadership		

a Number in () represents ranking in Faure *et al.*’s review.

b Number in () denotes number of sources including each theme in Monette *et al.*’s review.

## Discussion

The current study summarized and reported on the key features of principles, guidelines, frameworks and models for North–South research partnerships drawn from the academic, policy and practitioner domains. It endorses and extends the findings of two scoping reviews specific to global health, which were published in early 2021 (Faure *et al.*, 2021; Monette *et al.*, 2021). The recent publication of these reviews and the trend indicated by the publication dates of guidelines included in the current study are consistent with the growing momentum in global health to address issues of inequity between Northern and Southern stakeholders and to improve how research partnerships work.

In contrast to Faure *et al.’s* (2021) and Monette *et al.’s* (2021) reviews, which focused on global health, the current study did not limit the search to any particular discipline. However, despite this, two fields strongly dominated the search results: global health and international development. This cannot be explained by selection bias alone since, although one of the databases searched specializes in health, the other two and Google have broad coverage. A possible interpretation is that practitioners and researchers in these fields are more acutely aware than those from other fields of the colonial roots of their disciplines and the need to challenge the systems and structures that perpetuate inequities.

A quarter of guidelines did not specify their target audience. A further third included a broad category of ‘all other stakeholders’ to mop up unspecified audiences alongside major stakeholder groups, such as researchers and funders. Imprecision in defining who the guidelines were designed for may reflect the broad applicability of principles of partnership but, in some cases, may imply a lack of critical engagement in how guidelines are operationalized in a real-world context. This would be consistent with claims that imprecision in defining partnership has contributed to a lack of progress in addressing partnership inequities (Crane, 2010; Gautier *et al.*, 2018). No guideline overtly distinguished between Northern and Southern audiences. In so doing, they avoided engaging with the intractable issues of imbalances in power, control, access to resources and capacity (Healey-Walsh *et al.*, 2019; Craveiro *et al.*, 2020), which have underpinned the development of many guidelines.

All guidelines were evidence-informed. Half drew on existing literature, including other guidelines. Two thirds combined multiple methods of research and consultation. More Northern participants were consulted during guideline development than Southern participants: this is consistent with the broader partnership discourse in which Southern perspectives on partnerships are under-represented (Bradley, 2007). A limitation of the review was that only English language publications were included, which may exacerbate the over-representation of Northern stakeholder perspectives.

The structure of guidelines varied considerably. Several guidelines used a lifecycle concept to highlight issues to be addressed during different phases of a partnership from initiation through to conclusion. Guidelines were mapped along a continuum from *values*  *based* to *activity*  *based*. Values-based guidelines emphasized relational constructs, such as fairness, respect, inclusion and humility, while *activity-based* guidelines were organized around concrete topics and actions. Faure *et al.* (2021) applied a similar distinction in their review of equity in international health collaborations describing these dimensions as *relational* and *structural*. While values-based guidelines may be more flexible and can be adapted to a wider variety of partnership arrangements, the strength of activity-based guidelines is that they address concrete issues and can be followed as a set of instructions for good practice. A number of the guidelines occupied the middle ground and combined values-based and activity-based components, enabling users to exploit the advantages of each.

A number of guidelines revolved around equity, or fairness, as a central construct, a finding also reflected in Monette *et al.’s* (2021), which described equity as ‘a shared vision, fundamental goal, or encompassing value’ (p. 9). Some guidelines referred to equity interchangeably with equality. These risks downplaying important structural imbalances often existing between Northern and Southern partners (Boum II *et al.*, 2018) since while partnerships may strive to be equitable, partners often do not have equal opportunities, resources and capacities. Whilst several guidelines embraced the construct of equity as a normative position, that is, for it to be right that partnerships are fair, there was little discussion about whether equitable partnerships deliver ‘better’ outcomes. Alba *et al.* (2020b) addressed the issue to some extent in their guideline for bridging research integrity with standards of global health epidemiology, but further exploration of whether fairer leads to better is needed and requires interrogation of what ‘good’ research is and whose opinions on this matter.

To summarize the content of the 22 guidelines included in this scoping review presented a challenge due to the volume of material and the diverse structure and style of the various guidelines. The summarizing process risked losing specificity through the abstraction of concepts. Nevertheless, the structured process that was followed of coding the content of guidelines and organizing codes resulted in an interpretable set of topics. A further step was taken to disaggregate the topics by the two major disciplines, which contributed guidelines to the scoping review: international development and global health. A comparison of the topics covered in guidelines from these disciplines showed that the three most prevalent topics were addressed equally by both fields. After this, there were some differences in the emphasis of guidelines from global health and international development, but, rather than focusing on the differences, the authors suggest that there is greater value in pooling the guidance from the two disciplines.

A comparison of the topics, combined across disciplines, from this review with the themes identified in reviews by Faure *et al.* (2021) and Monette *et al.* (2021) identified substantial overlap. Faure *et al.*’s scoping review focused on experiences and perspectives of equity in international health collaborations and included qualitative empirical studies, opinion pieces and editorials. The authors reflected in their discussion on the need to expand the review of the literature to encompass frameworks and guidelines. The current study sought to address this issue by seeking out principles, guidelines, frameworks and models of partnership. The scoping review by Monette *et al.* (2021) published in March 2021 sought to elicit the principles of ‘good’ global health research partnerships. It was informed by nine documents, six of which were also included in this review. The concordance of themes from across these three complementary reviews form a solid base from which to focus efforts to improve how partnerships work. However, there is a risk that guidance becomes self-referencing and fails to identify blind spots arising from the under-representation of Southern stakeholder perspectives in guideline development. Furthermore, while the number of separate guidelines addressing a particular topic area provides an indication of its importance, individual guidelines typically presented topics as a package and not hierarchically. Further work is needed to explore the interplay between topics, whether some matter more than others and how this varies from the perspective of different stakeholder groups. This would be valuable for focusing efforts where partnership resources and time are limited and to provide leverage in negotiating funder and institutional policies towards more equitable partnership arrangements. Other issues to explore in future studies include: how Southern institutions can best advocate for equity in partnerships, what else funders should do to promote fairness and how best to share exemplars of good partnership practice.

## Conclusion

There is no shortage of guidance for North–South research partnerships, and considerable agreement on the key areas where attention needs to be paid in order for partnerships to be fair. However, Northern perspectives dominate the guidance and further exploration of what matters to Southern stakeholders is needed. Work to explore how guidelines are used, whether they make any difference and to examine the relationship between the quality of partnerships and the quality of research generated would take the field forward.

Furthermore, challenges to the foundations of global health, an increase in funding channelled directly to the global South and the maturation of world-class Southern research institutions coalescing with truly global challenges, such as COVID-19 and climate change, are likely to stimulate new partnership dynamics to take hold.

## Supplementary Material

czac008_Supp

## Data Availability

The data underlying this article are available in the article and in its online supplementary material.

## References

[R1] 3rd World Conference on Research Integrity . 2013. Montreal statement on research integrity in cross-boundary research collaborations. 5–8 May 2013. Montreal.

[R2] Afsana K, Habte D, Hatfield J, Murphy J, Neufield V. 2009. *Partnership Assessment Toolkit*. Ottawa, Canada: Canadian Coalition for Global Health Research.

[R3] Alba S, Lenglet A, Verdonck K et al. 2020a. Bridging research integrity and global health epidemiology (BRIDGE) guidelines: explanation and elaboration. *BMJ Global Health* 5: e003237. 10.1136/bmjgh-2020-003237 PMC7594201 33115860

[R4] Alba S, Verdonck K, Lenglet A et al. 2020b. Bridging research integrity and global health epidemiology (BRIDGE) statement: guidelines for good epidemiological practice. *BMJ Global Health* 5: e003236. 10.1136/bmjgh-2020-003236 PMC7594207 33115859

[R5] Arksey H, O’Malley L. 2005. Scoping studies: towards a methodological framework. *International Journal of Social Research Methodology* 8: 19–32.

[R6] Association of Universities and Colleges of Canada . 2013. Innovative North South Partnerships: synthesis of findings. Case Studies.

[R7] Binka F . 2005. Editorial: North-South research collaborations: a move towards a true partnership? *Tropical Medicine and International Health* 10: 207–9. 15730502 10.1111/j.1365-3156.2004.01373.x

[R8] Boum II Y, Burns BF, Siedner M et al. 2018. Advancing equitable global health research partnerships in Africa. *BMJ Global Health* 3: e000868. 10.1136/bmjgh-2018-000868 PMC6112391 30167335

[R9] Bradley M . 2007. North–south research partnerships: challenges, responses and trends—a literature review and annotated bibliography. IDRC Canadian Partnerships Working Papers (ed.) *Working Paper 1*.

[R10] Bradley M . 2008. On the agenda: North–South research partnerships and agenda-setting processes. *Development in Practice* 18: 673–85.

[R11] Bradley M . 2017. Whose agenda? North South research partnerships. In: Mougeot L. (ed.) *Putting Knowledge to Work: Collaborating, Influencing and Learning for International Development*. Rugby, UK: Practical Action Publishing.

[R12] Canadian Coalition for Global Health Research . 2015. CCGHR principles for global health research.

[R13] Carbonnier G, Kontinen T. 2014. North-South research partnership: academia meets development? European Assocation of Development Reseach and Training Institutes.

[R14] Coloma J, Harris E. 2009. From construction workers to architects: developing scientific research capacity in low-income countries. *PLoS Biology* 7: e1000156. 10.1371/journal.pbio.1000156 PMC2704958 19621063

[R15] Corbin H, Mittelmark MB, Lie GT. 2012. Scaling-up and rooting-down: a case study of North-South partnerships for health from Tanzania. *Global Health Action* 5: 18369. 10.3402/gha.v5i0.18369 PMC3387362 22761602

[R16] Cornish H, Fransman J, Newman K. 2017. Rethinking research partnerships: discussion guide and toolkit.

[R17] Costello A, Zumla A. 2000. Moving to research partnerships in developing countries. *BMJ* 321: 827–9. 11009530 10.1136/bmj.321.7264.827 PMC1118627

[R18] Crane J . 2010. Unequal ‘partners’. AIDS, academia, and the rise of global health. *Behemoth* 3: 78–97.

[R19] Craveiro I, Carvalho A, Ferrinho P. 2020. “Get us partnerships!” - a qualitative study of Angolan and Mozambican health academics’ experiences with North/South partnerships. *Globalization and Health* 16. doi: 10.1186/s12992-020-00562-7. PMC7161017 32295611

[R20] Dodson J . 2017. *Building Partnerships of Equals: The Role of Funders in Equitable and Effective International Development Collaborations*. London, United Kingdom: UK Collaborative on Development Sciences.

[R21] Edejer TTT . 1999. North-South research partnerships: the ethics of carrying out research in developing countries. *BMJ* 319: 438–41. 10445930 10.1136/bmj.319.7207.438 PMC1127046

[R22] ESPA Directorate . 2018. *Research for Development Impact: The Role of Equitable Partnerships: Policy and Practice Briefing*. Edinburgh, Scotland, United Kingdom: Ecosystem Services for Poverty Alleviation (ESPA).

[R23] Faure MC, Munung NS, Ntusi NAB, Pratt B, De Vries J. 2021. Mapping experiences and perspectives of equity in international health collaborations: a scoping review. *International Journal for Equity in Health* 20: 1–13. 33422065 10.1186/s12939-020-01350-w PMC7796532

[R24] Feld A, Kreimer P. 2019. Scientific co-operation and centre-periphery relations: attitudes and interests of European and Latin American scientists. *Tapuya: Latin American Science, Technology and Society* 2: 149–75.

[R25] Forti S . 2005. *Building a Partnership for Research in Global Health: An Analytical Framework*. Canada: Canadian Coalition for Global Health Research Research.

[R26] Franzen SRP, Chandler C, Lang T. 2017. Health research capacity development in low and middle income countries: reality or rhetoric? A systematic meta-narrative review of the qualitative literature. *BMJ Open* 7: e012332. 10.1136/bmjopen-2016-012332 PMC5278257 28131997

[R27] Gaillard JF . 1994. North-South research partnership: is collaboration possible between unequal partners? *Knowledge and Policy* 7: 31–63.

[R28] Gautier L, Sieleunou I, Kalolo A. 2018. Deconstructing the notion of “global health research partnerships” across Northern and African contexts. *BMC Medical Ethics* 19: 49. 10.1186/s12910-018-0280-7 PMC6019997 29945595

[R29] González-Alcaide G, Menchi-Elanzi M, Nacarapa E, Ramos-Rincón J-M. 2020. HIV/AIDS research in Africa and the Middle East: participation and equity in North-South collaborations and relationships. *Globalization and Health* 16. doi: 10.1186/s12992-020-00609-9. PMC7499968 32943058

[R30] Harrison E . 2002. ‘The problem with the locals’: partnership and participation in Ethiopia. *Development and Change* 33: 587–610.

[R31] Healey-Walsh J, Stuart-Shor E, Muchira J. 2019. Through the lens of postcolonial theory. *Nursing Education Perspectives* 40: 270–7. 31436689 10.1097/01.NEP.0000000000000556

[R32] Hommes F, Monzó HB, Ferrand RA et al. 2021. The words we choose matter: recognising the importance of language in decolonising global health. *The Lancet Global Health* 9: e897–8. 34143986 10.1016/S2214-109X(21)00197-2

[R33] Jentsch B, Pilley C. 2003. Research relationships between the South and the North: Cinderella and the ugly sisters? *Social Science & Medicine* 57: 1957–67. 14499518 10.1016/s0277-9536(03)00060-1

[R34] Kennedy HP, Renfrew MJ, Madi BC, Opoku D, Thompson JB. 2006. The conduct of ethical research collaboration across international and culturally diverse communities. *Midwifery* 22: 100–7. 16698154 10.1016/j.midw.2006.03.001

[R35] Kunert KJ, Botha A-M, Oberholster PJ et al. 2020. Factors facilitating sustainable scientific partnerships between developed and developing countries. *Outlook on Agriculture* 49: 204–14. 32981973 10.1177/0030727020939592 PMC7491427

[R36] Larkan F, Uduma O, Lawal SA, Van Bavel B. 2016. Developing a framework for successful research partnerships in global health. *Globalization and Health* 12. doi: 10.1186/s12992-016-0152-1. PMC4859962 27154550

[R37] Leffers J, Mitchell E. 2011. Conceptual model for partnership and sustainability in global health. *Public Health Nursing* 28: 91–102. 21198819 10.1111/j.1525-1446.2010.00892.x

[R38] Matenga TFL, Zulu JM, Corbin JH, Mweemba O. 2019. Contemporary issues in north–south health research partnerships: perspectives of health research stakeholders in Zambia. *Health Research Policy and Systems* 17. doi: 10.1186/s12961-018-0409-7. PMC6334387 30646902

[R39] Migot-Adholla S, Warner M. 2005. North-South research partnerships: a guidance note on the partnering process London Overseas Development Institute.

[R40] Mommers C, van Wessel M. 2009. Structures, values, and interaction in field-level partnerships: the case of UNHCR and NGOs. *Development in Practice* 19: 160–72.

[R41] Monette EM, McHugh D, Smith MJ et al. 2021. Informing ‘good’ global health research partnerships: a scoping review of guiding principles. *Global Health Action* 14: 1892308. 10.1080/16549716.2021.1892308 PMC7954413 33704024

[R42] Mony PK, Kurpad A, Vaz M. 2005. Capacity building in collaborative research is essential. *BMJ* 331: 843–44. 10.1136/bmj.331.7520.843-b PMC1246129 16210297

[R43] Munn Z, Peters MDJ, Stern C et al. 2018. Systematic review or scoping review? Guidance for authors when choosing between a systematic or scoping review approach. *BMC Medical Research Methodology* 18: 143. 10.1186/s12874-018-0611-x PMC6245623 30453902

[R44] Murphy J, Hatfield J, Afsana K, Neufeld V. 2015. Making a commitment to Ethics in global health research partnerships: a practical tool to support ethical practice. *Journal of Bioethical Inquiry* 12: 137–46. 25648123 10.1007/s11673-014-9604-6

[R45] Newman K, Bharadwaj S, Fransman J. 2019. Rethinking research impact through principles for fair and equitable partnerships. *IDS Bulletin* 50: 21–42. doi: 10.19088/1968-2019.104.

[R46] Page MJ, Mckenzie JE, Bossuyt PM et al. 2021. The PRISMA 2020 statement: an updated guideline for reporting systematic reviews. *BMJ* 372: n71. 10.1136/bmj.n71 PMC8005924 33782057

[R47] RAWOO . 1999. *North-South Research Partnerships: Issues and Challenges Trivandrum Expert Meeting*. The Hague: Netherlands Development Assistance Research Council (RAWOO).

[R48] RAWOO . 2001. *Balancing Ownership and Partnership in Development Research: Review of 1999 and 2000*. The Hague: RAWOO.

[R49] Research Fairness Initiative . 2018. RFI Guides. Council on Health Research for Development. https://rfi.cohred.org/rfi-guides/, accessed 10 August 2021.

[R50] Rethinking Research Collaborative . 2018. Promoting fair and equitable research partnerships to respond to global challenges: recommendations to the UKRI UK research and innovation.

[R51] Sawyerr A . 2004. African universities and the challenge of research capacity development. *Journal of Higher Education in Africa* 2: 213–42.

[R52] Stöckli B, Wiesmann U, Lys J-A. 2018. *A Guide for Transboundary Research Partnerships, 11 Principles, 3rd Edition (1st Edition 2012)*. 3rd edn. Bern, Switzerland: Swiss Commission for Research Partnerships with Developing Countries (KFPE).

[R53] Taylor LA, Berg DN. 2019. The challenge of mutual disclosure in global health partnerships. *Perspectives in Biology and Medicine* 62: 657–74. 31761799 10.1353/pbm.2019.0038

[R54] Trust . 2018. Global code of conduct for research in resource poor settings. Trust.

[R55] United Nations . 2020. *Goal 17: Strengthen the Means of Implementation and Revitalize the Global Partnership for Sustainable Development* [Online]. https://www.un.org/sustainabledevelopment/globalpartnerships/, accessed 30 August 2021.

[R56] Viergever RF, Olifson S, Ghaffar A, Terry RF. 2010. A checklist for health research priority setting: nine common themes of good practice. *Health Research Policy and Systems* 8. doi: 10.1186/1478-4505-8-36. PMC3018439 21159163

[R57] Walsh A, Brugha R, Byrne E. 2016. “The way the country has been carved up by researchers”: ethics and power in north–south public health research. *International Journal for Equity in Health* 15. 10.1186/s12939-016-0488-4 PMC5153695 27955670

[R58] White MT . 2007. A right to benefit from international research: a new approach to capacity building in less-developed countries. *Accountability in Research* 14: 73–92. 17844784 10.1080/08989620701290341

[R59] White SC . 2020. A space for unlearning? A relational perspective on North–South development research. *The European Journal of Development Research* 32: 483–502.

