# Supplementary material for: What makes working together work? A scoping review of the guidance on North–South research partnerships
Source: Health Policy Plan. 2022 Jan 28;37(4):523–34. doi: 10.1093/heapol/czac008 (PMC9006068; doi:10.1093/heapol/czac008)
Supplement: czac008_Supp [file czac008_supp.zip › Supplementary table 1.docx]

**Supplementary table 1: Topics addressed by each partnership guideline**

|  | **Roles, respons-ibilities & ways of working** | **Capacity strengthening** | **Resource contributions** | **Motivation & goals** | **Agenda setting & study design** | **Governance structures, institutional agreements** | **Dissemination** | **Respect for affected populations, including local relevance** | **Data handling and owner-ship** | **Funding** | **Long term commit-ments** | **Acknow-ledging power dynamics & inequal-ities** | **Trust** | **M&E** | **Ethical approvals** | **Shared benefits** | **Justification for research** | **Appreciation of context** | **Admin support** | **Closure plans** | **Leader-ship** |
| --- | --- | --- | --- | --- | --- | --- | --- | --- | --- | --- | --- | --- | --- | --- | --- | --- | --- | --- | --- | --- | --- |
| Rank | 1 | 2 | 3 | =3 | 5 | =5 | =7 | =7 | =7 | 10 | =10 | 12 | =12 | 14 | =14 | =14 | 17 | 18 | =19 | =19 | =19 |
| Count | 18 | 15 | 14 | 14 | 11 | 11 | 10 | 10 | 10 | 8 | 8 | 7 | 7 | 6 | 6 | 6 | 5 | 4 | 2 | 2 | 2 |
| Afsana et al, 2009 | ✓ | ✓ | ✓ | ✓ | ✓ | ✓ | ✓ | ✓ | ✓ |  | ✓ |  |  | ✓ | ✓ |  |  |  |  | ✓ |  |
| Alba et al, 2020 |  | ✓ |  |  | ✓ | ✓ | ✓ | ✓ | ✓ |  |  |  |  |  | ✓ |  | ✓ |  |  |  |  |
| Association of Universities and Colleges of Canada/IDRC 2013 | ✓ | ✓ | ✓ | ✓ | ✓ |  |  |  |  |  |  | ✓ | ✓ |  |  |  |  |  |  |  | ✓ |
| Canadian Coalition for Global Health Research, 2015 | ✓ |  |  |  |  |  | ✓ | ✓ | ✓ |  | ✓ | ✓ | ✓ |  |  | ✓ |  | ✓ |  |  |  |
| Carbonnier & Kontinen, 2014 |  |  |  | ✓ | ✓ |  |  |  |  |  | ✓ |  |  |  |  | ✓ |  |  |  |  |  |
| Cornish, Fransman & Newman, 2017 | ✓ | ✓ | ✓ | ✓ | ✓ |  | ✓ |  | ✓ | ✓ |  | ✓ |  | ✓ | ✓ |  |  | ✓ |  | ✓ |  |
| Costello & Zumla, 2000 | ✓ | ✓ |  |  |  | ✓ |  | ✓ |  |  |  |  | ✓ |  |  |  |  |  |  |  |  |
| Dodson, 2017 |  | ✓ | ✓ |  | ✓ |  |  |  |  | ✓ |  |  |  |  |  |  |  | ✓ |  |  |  |
| Ecosystem Services for Poverty Alleviation (ESPA), 2018 | ✓ |  | ✓ | ✓ | ✓ |  |  |  |  |  |  |  |  |  |  | ✓ |  |  |  |  |  |
| Faure et al, 2021 | ✓ | ✓ | ✓ |  |  | ✓ | ✓ | ✓ | ✓ | ✓ |  | ✓ | ✓ |  |  |  |  |  |  |  |  |
| Gaillard, 1994 | ✓ | ✓ | ✓ | ✓ | ✓ |  | ✓ |  |  | ✓ | ✓ |  |  | ✓ |  |  |  |  |  |  |  |
| Kennedy et al, 2006 | ✓ |  | ✓ | ✓ |  | ✓ | ✓ | ✓ | ✓ | ✓ |  | ✓ |  | ✓ | ✓ |  | ✓ |  |  |  |  |
| Larkan et al, 2016 | ✓ |  | ✓ | ✓ |  | ✓ |  |  |  |  |  | ✓ | ✓ |  |  |  |  |  | ✓ |  | ✓ |
| Leffers & Mitchell, 2011 | ✓ | ✓ |  | ✓ |  |  |  |  |  |  |  |  |  |  |  |  |  |  |  |  |  |
| Montreal Statement on Research Integrity, 2013 | ✓ |  | ✓ | ✓ |  | ✓ | ✓ |  | ✓ |  |  |  | ✓ | ✓ |  | ✓ | ✓ |  |  |  |  |
| Newman & Fransman, 2019/ Rethinking Research Collaborative, 2018 | ✓ | ✓ | ✓ | ✓ |  | ✓ |  | ✓ |  |  | ✓ |  |  |  |  |  |  | ✓ |  |  |  |
| Overseas Development Institute (ODI), 2005 | ✓ | ✓ |  | ✓ |  | ✓ |  |  |  |  | ✓ |  |  |  |  |  |  |  |  |  |  |
| RAWOO, 1999 | ✓ | ✓ |  | ✓ | ✓ |  |  |  |  |  |  |  |  |  |  |  |  |  |  |  |  |
| Research Fairness Initiative, 2018 | ✓ | ✓ | ✓ |  | ✓ | ✓ |  | ✓ | ✓ | ✓ | ✓ | ✓ |  |  | ✓ |  | ✓ |  | ✓ |  |  |
| Swiss Commission for Research Partnerships with Developing Countries (KFPE), 2018 | ✓ | ✓ | ✓ | ✓ | ✓ | ✓ | ✓ | ✓ | ✓ | ✓ | ✓ |  |  | ✓ |  | ✓ | ✓ |  |  |  |  |
| Taylor & Berg, 2019 |  |  |  |  |  |  |  |  |  |  |  |  | ✓ |  |  |  |  |  |  |  |  |
| TRUST, 2018 | ✓ | ✓ | ✓ |  |  |  | ✓ | ✓ | ✓ | ✓ |  |  |  |  | ✓ | ✓ |  |  |  |  |  |
